# Domain‐Shuffling in the Evolution of Cyclostomes and Gnathostomes

**DOI:** 10.1002/jez.b.23282

**Published:** 2024-12-04

**Authors:** Hirofumi Kariyayama, Takeshi Kawashima, Hiroshi Wada, Haruka Ozaki

**Affiliations:** ^1^ Graduate School of Comprehensive Human Sciences University of Tsukuba Tsukuba Ibaraki Japan; ^2^ Department of Genetics The Graduate University for Advanced Studies, SOKENDAI Mishima Shizuoka Japan; ^3^ National Institute of Genetics Mishima Shizuoka Japan; ^4^ Institute of Life and Environmental Sciences University of Tsukuba Tsukuba Ibaraki Japan; ^5^ Bioinformatics Laboratory, Institute of Medicine University of Tsukuba Tsukuba Ibaraki Japan; ^6^ Center for Artificial Intelligence Research University of Tsukuba Tsukuba Ibaraki Japan

**Keywords:** comparative genomics, cyclostome, domain‐shuffling origin gene, gnathostome, vertebrate

## Abstract

Vertebrates acquired various novel traits that were pivotal in their morphological evolution. Domain shuffling, rearrangements of functional domains between genes, is a key molecular mechanism in deuterostome evolution. However, comprehensive studies focusing on early vertebrates are lacking. With advancements in genomic studies, the genomes of early vertebrate groups and cyclostomes are now accessible, enabling detailed comparative analysis while considering the timing of gene acquisition during evolution. Here, we compared 22 metazoans, including four cyclostomes, to identify genes containing novel domain architectures acquired via domain‐shuffling (DSO‐Gs), in the common ancestor of vertebrates, gnathostomes, and cyclostomes. We found that DSO‐Gs in the common ancestor of vertebrates were associated with novel vertebrate characteristics and those in the common ancestor of gnathostomes correlated with gnathostome‐specific traits. Notably, several DSO‐Gs acquired in common ancestors of vertebrates have been linked to myelination, a distinct characteristic of gnathostomes. Additionally, in situ hybridization revealed specific expression patterns for the three vertebrate DSO‐Gs in cyclostomes, supporting their potential functions. Our findings highlight the significance of DSO‐Gs in the emergence of novel traits in the common ancestors of vertebrates, gnathostomes, and cyclostomes.

## Graphical abstract

We addressed the potential roles of domain‐shuffling origin genes (DSO‐Gs) in the evolution of early vertebrates. Through comparative genome analysis of 22 metazoans, including four cyclostomes, we identified DSO‐Gs before and after the divergence of cyclostomes and gnathostomes, and suggest domain shuffling as a key mechanism in vertebrate early evolution.

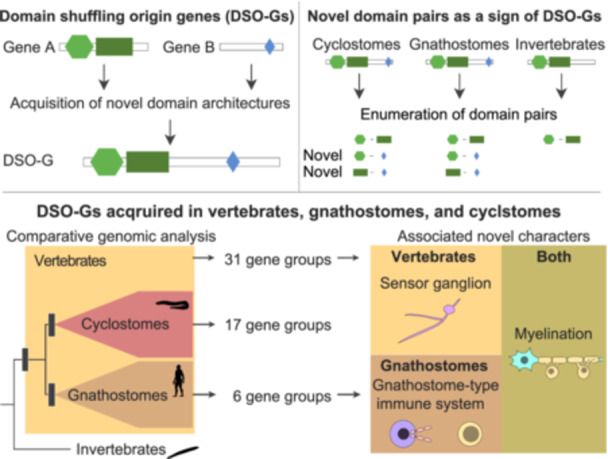

## 1 Introduction

Vertebrates have acquired various novel traits during their early evolution (Janvier 2003; Romer and Parsons 1977; Young 1962). These novel characteristics contribute to the vertebrate diversity. For example, various tissues derived from the neural crest cells and cartilaginous tissues support vertebrate bodies and regulate their motility.

The evolution of novel traits can be attributed to genetic modifications, leading to changes in gene expression and the acquisition of novel genes (Brakefield 2006; Kaessmann 2010). Genetic modifications include mutations, gene duplications, horizontal gene transfer, genome rearrangements, and de novo gene births (Koonin 2005; Long et al. 2003). Domain shuffling is a form of genome rearrangements characterized by the recruitment of functional domains from other genes to one gene (Patthy 1999) via exon shuffling and transposable elements (Babushok, Ostertag, and Kazazian 2007; Patthy 2003). Consequently, genes with novel domain architectures acquired by domain‐shuffling, we call domain‐shuffling origin genes (DSO‐Gs) which have new combinations of functional domains, presumably contributing to new gene functions (Patthy 2003). A previous study compared gene models of 20 organisms, identified many examples of DSO‐Gs and showed that domain shuffling contributed to the evolution of metazoa (Ekman, Björklund, and Elofsson 2007; Kawashima et al. 2009). However, comprehensive studies focusing on domain shuffling in early vertebrates are lacking, partly because of the lack of available genomes of early vertebrate groups, such as cyclostomes.

Cyclostomes are a lineage of vertebrates that comprise lampreys and hagfish and a sister group of gnathostomes (jawed vertebrates) that include most vertebrates. While cyclostomes share traits with gnathostomes, such as neural crest cells, image‐forming vision, and thyroid glands, they do not share other traits such as jaw morphology, paired appendages, myelin sheaths, sympathetic ganglia, and gnathostome‐type adaptive immunity systems (Hardisty 1979, 1982). While the cyclostomes may lose these characteristics secondary, the absence of these gnathostome‐specific traits in cyclostomes is likely an ancestral characteristic of vertebrates. Thus, multiple novel characteristics appeared stepwise during the early evolution of vertebrates.

**Figure 1:**
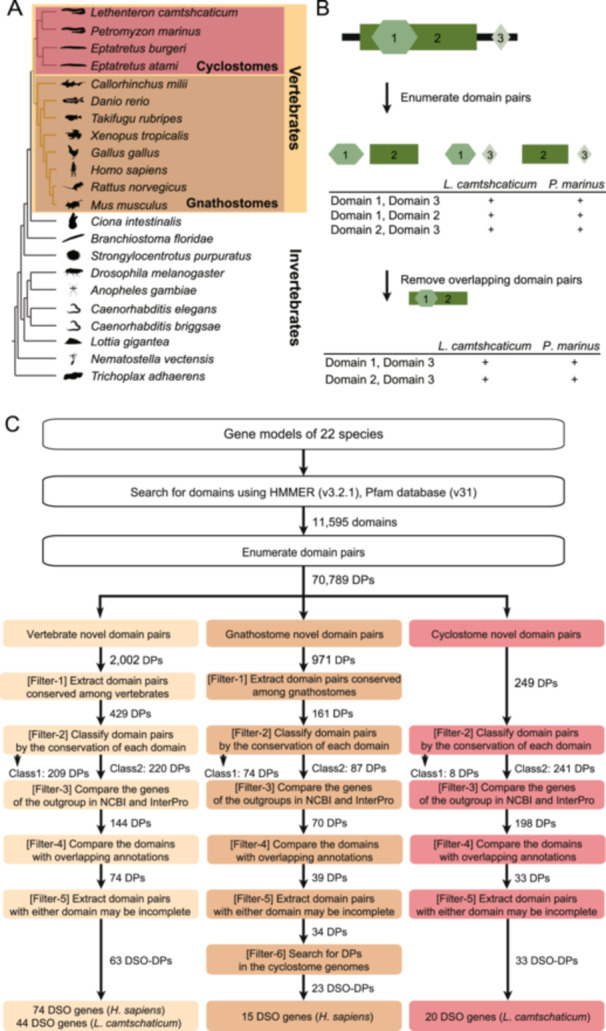
An overview of the identification of domain‐shuffling origin genes (DSO‐Gs). (A) The Phylogenetic tree of the species included in the domain‐shuffling analysis. Animal diagrams were created on PhyloPic (http://phylopic.org). (B) A schematic of the enumeration of domain pairs (DPs) from domains on a gene. (C) The flow of DSO‐G extraction and the number of DPs at each step.

Focusing on the divergence between cyclostomes and gnathostomes will provide valuable insights into the evolution of key vertebrate traits and the underlying genetic mechanisms. For example, investigating the DSO‐Gs that occur before and after the divergence of cyclostomes and gnathostomes can elucidate the significance of domain shuffling in vertebrate evolution. Recently, the genome sequences of cyclostomes (jawless vertebrates) have been made available, including *Lethenteron camtschaticum* (Mehta et al. 2013), gene models provided by Kodota et al. (Kadota et al. 2017), *Petromyzon marinus* (Smith et al. 2013, 2018; Timoshevskaya et al. 2023), *Eptatretus burgeri* (Pascual‐Anaya et al. 2018), and *Eptatretus atami* (Marlétaz et al. 2024). These cyclostome genomes allow us to associate the timing of the DSO‐G acquisition with novel trait acquisitions before and after the divergence of gnathostomes and cyclostomes (Figure 1A).

In this study, we compared 22 metazoa genomes and identified DSO‐Gs with novel domain architectures acquired via domain shuffling, both at the timing before and after the divergence of gnathostomes and cyclostomes (Figure 1A). We found that vertebrate DSO‐Gs were linked to novel characteristics of vertebrates, and that gnathostome DSO‐Gs were related to novel characteristics of gnathostomes. Our results highlight the potential impact of domain shuffling on the evolution of defining traits in common ancestors of vertebrates, gnathostomes, and cyclostomes.

## 2 Materials and Methods

### 2.1 Definition of Terminology

In this study, we define several types of domain pairs as follows. A *domain pair* refers to a pair of functional domains on a gene. A *lineage‐specific domain pair* is defined as a domain pair which is found from only one lineage and is not found from its outgroup metazoan species. A *domain‐shuffling origin domain pair (DSO‐DP)* means a domain pair which is assumed to have been acquired by a domain‐shuffling event (see Section 2.4). To indicate genes and gene groups containing DSO‐DPs, we define the terms: *domain‐shuffling origin gene (DSO‐G)* referring to a gene having a DSO‐DP, and *domain‐shuffling origin gene group (DSO‐GG)* which denotes a gene group which is assumed to have appeared through a common domain‐shuffling event.

### 2.2 Enumeration of Domain Pairs in Animals

Domain shuffling is a theory where a new gene with a new domain combination has emerged in the genome (Patthy 2003). Thus, to extract DSO‐Gs, we should identify a single gene encompassing two different functional domains which had never coexisted in the other taxonomic clades. To detect such domain combinations, we here focused on a pair of functional domains in a gene (Chothia et al. 2003; Kawashima et al. 2009), which is hereafter referred to as a domain pair.

We used gene models of 22 metazoans, including four cyclostomes, Arctic lamprey (*L*. *camtschaticum*), sea lamprey (*P*. *marinus*), hagfish (*E*. *burgeri*), brown hagfish (*E*. *atami*), eight gnathostomes, human (*Homo sapiens*), mouse (*Mus musculus*), rat (*Rattus norvegicus*), chicken (*Gallus gallus*), frog (*Xenopus tropicalis*), zebrafish (*Danio rerio*), fugu (*Takifugu rubripes*), and ghost shark (*Callorhinchus milii*), and 10 invertebrates, *Trichoplax adhaerens*, sea anemone (*Nematostella vectensis*), two nematodes (*Caenorhabditis elegans* and *Caenorhabditis briggasae*), mosquito (*Anopheles gambiae*), fruit fly (*Drosophila melanogaster*), limpet (*Lottia gigantea*), sea urchin (*Strongylocentrotus purpuratus*), amphioxus (*Branchiostoma floridae*), and ascidian (*Ciona intestinalis*), from the genome databases (Supporting Information S2: Table S1). To identify functional domains in the translated sequences of the gene models, we used the HMMER3 program version 3.2.1 (Mistry et al. 2013), a software for searching homologous motifs using the hidden Markov model with Pfam‐A entries from the Pfam database version 31 (Finn et al. 2016). We set an E‐value threshold of < 1e−3 to select the hits by HMMER3 as candidate functional domains.

Domain pairs were extracted using the method proposed by Kawashima et al. (Kawashima et al. 2009), with some modifications. We did not distinguish the order of the domain pairs in a gene. All domain pairs were enumerated as follows (1, 2) (Figure 1B). (1) We enumerated all unique pairs of functional domains in each gene for each species. (2) We compiled a binary matrix representing the presence or absence of each domain pair across species.

To exclude cases where the detected domain pairs overlapped with the position of a gene, we proceeded as follows (Figure 1B). First, we created a binary matrix showing the presence or absence of domain pairs where one domain was covered by other domains. Finally, we removed domain pairs in which the two domains overlapped according to the following criteria: the overlapping domain pair relationship was conserved among all species having the domain pair. As a result, we compiled a list of 70,789 domain pairs used in the downstream analysis.

### 2.3 Extraction of Lineage‐Specific Domain Pairs

We extracted lineage‐specific domain pairs that were detected only in each clade of deuterostomes, chordates, olfactores, vertebrates, cyclostomes, gnathostomes, euteleostomi, and tetrapods (Supporting Information S3: Figure S1). Here, we defined lineage‐specific domain pairs as domain pairs shared by at least two species in a certain lineage, one of which was included in the first divided lineage and the others were included in a sister group of the lineage, and were not found from outgroup species. For instance, we defined vertebrate‐specific domain pairs as domain pairs shared with at least a cyclostome and a gnathostome. Gnathostome‐specific domain pairs were defined as domain pairs shared with Chondrichthyes (ghost shark in this study) and at least one of the Euteleostomi. We also defined cyclostome‐specific domain pairs as those shared by at least one species of the lampreys and the hagfishes. Under these criteria, we listed 2,002, 971, and 249 lineage‐specific domain pairs for vertebrates, gnathostomes, and cyclostomes, respectively (Supporting Information S3: Figure S1). However, it is important to note that our method may underestimate or overestimate the number of lineage‐specific domain pairs owing to convergent acquisition or secondary loss.

### 2.4 Identification of DSO‐DPs and DSO‐Gs

To identify the genes acquired via domain shuffling that contributed to the emergence of novel characters, we filtered vertebrate‐, gnathostome‐, and cyclostome‐specific domain pairs to extract their corresponding DSO‐Gs (Figure 1C).

First, we extracted the highly conserved domain pairs in each lineage (Filter 1). For vertebrates, we identified DSO‐DPs conserved among all gnathostomes and at least one cyclostome from vertebrate‐specific domain pairs. For gnathostomes, we extracted DSO‐DPs conserved among all gnathostomes from gnathostome‐specific domain pairs. For cyclostomes, we extracted DSO‐DPs conserved both at least one of the hagfishes and the lampreys from cyclostome‐specific domain pairs.

Secondly, we then classified the vertebrate‐, gnathostome‐, and cyclostome‐specific domain pairs into Class 1 and Class 2 domain pairs by the conservation of domains (Filter 2) to distinguish the lineage‐specific domain pairs considered having to be acquired by domain shuffling from the others. We used the domain pair classification described by Kawashima et al. (Kawashima et al. 2009). We classified a lineage‐specific domain pair as Class 1 if either domain was shared only within a certain lineage. For Class 1, we could not distinguish between the lineage‐specific domain pairs acquired via sequence substitutions or domain shuffling. Alternatively, we classified a lineage‐specific domain pair as Class 2 if both domains were shared with the outgroup animals. In Class 2, domain pairs were more likely to be acquired via domain shuffling than substitutions. Consequently, we identified 220 vertebrate‐specific domain pairs, 87 gnathostome‐specific domain pairs, and 241 cyclostome‐specific domain pairs as Class 2. We further classified Class 2 domain pairs into DSO‐DPs and others by conservation with outgroups and truncation of domains (Filters 3, 4, and 5). Additionally, we examined the presence of the regions corresponding to the gnathostome DSO gene candidates in cyclostome genomes using BLAST (Filter 6). A detailed description is provided in Appendix A. (Supporting Information S1). Finally, we classified 63 domain pairs as vertebrate DSO‐DPs (Supporting Information S2: Table S2). Similarly, 23 domain pairs were classified as gnathostome DSO‐DPs (Supporting Information S2: Table S3). 33 domain pairs were identified as cyclostome DSO‐DPs (Supporting Information S2: Table S4).

### 2.5 Grouping of DSO‐Gs Into DSO Gene Groups (DSO‐GGs)

To estimate the number of gene acquisitions by domain‐shuffling events, we defined DSO‐GGs which were assumed to originate from the same domain‐shuffling events. First, we merged the DSO‐Gs corresponding to each DSO‐DP into groups (initial DSO‐GGs). Next, if the two initial DSO‐GGs comprised the same genes, we merged them. Subsequently, if the two DSO‐DPs were nearly identical, the two corresponding initial DSO‐GGs were further merged into a single DSO‐GG. Most DSO‐Gs were single DSO‐GGs, while a few were multiple DSO‐GGs (Tables 1 and 3). Finally, we identified 31 gene groups from vertebrate DSO‐Gs (VGGs, Table 1), namely VGG, with a unique serial number for each gene group's name. We also categorized gnathostome DSO‐Gs into six gene groups, namely gnathostome DSO‐GGs (GGGs, Table 2), and cyclostome DSO‐GGs into 17 gene groups, namely cyclostome DSO‐GGs (CGGs, Table 3). The headings in Tables 1, 2, 3 clearly show the three relationships among DSO‐G, DSO‐GG, and DSO‐DP in this manuscript.

**Table 1:** The list of the vertebrate DSO‐DPs and genes.

DSO‐GGs	Represented DSO‐Gs		DSO‐DPs				Other DSO‐Gs
(=Gene Group ID)	Description	ENSEMBL gene ID	Domain 1		Domain 2		(=Gene IDs^a^)
VGG01	Angiotensin I converting enzyme 2	ENSG00000130234	PF01401	Peptidase_M2	PF16959	Collectrin	
VGG02	Attractin like 1	ENSG00000107518	PF00059	Lectin_C	PF01437	PSI	ENSG00000088812
			PF00059	Lectin_C	PF17205	PSI_integrin	
VGG03	Carboxypeptidase X, M14 family member 1	ENSG00000088882	PF00246	Peptidase_M14	PF00754	F5_F8_type_C	ENSG00000121898, ENSG00000106624
			PF00754	F5_F8_type_C	PF13620	CarboxypepD_reg	ENSG00000121898, ENSG00000106624
			PF00754	F5_F8_type_C	PF13715	CarbopepD_reg_2	ENSG00000106624
VGG04	Cartilage acidic protein 1	ENSG00000095713	PF01839	FG‐GAP	PF07645	EGF_CA	
			PF07593	UnbV_ASPIC	PF07645	EGF_CA	
			PF07645	EGF_CA	PF13517	VCBS	
VGG05	Coagulation factor II, thrombin	ENSG00000180210	PF00051	Kringle	PF00594	Gla	
VGG06	Discs large MAGUK scaffold protein 1	ENSG00000075711	PF09058	L27_1	PF10608	MAGUK_N_PEST	ENSG00000132535, ENSG00000150672
VGG07	Elastin microfibril interfacer 3	ENSG00000183798	PF04582	Reo_sigmaC	PF07546	EMI	ENSG00000132205
VGG08	F‐box protein 9	ENSG00000112146	PF00646	F‐box	PF04212	MIT	
			PF04212	MIT	PF12937	F‐box‐like	
VGG09	HtrA serine peptidase 3	ENSG00000170801	PF00050	Kazal_1	PF13365	Trypsin_2	ENSG00000166033
			PF00089	Trypsin	PF00219	IGFBP	ENSG00000166033, ENSG00000169495
			PF00219	IGFBP	PF00595	PDZ	ENSG00000166033, ENSG00000169495
			PF00219	IGFBP	PF10459	Peptidase_S46	ENSG00000166033, ENSG00000169495
			PF00219	IGFBP	PF13180	PDZ_2	ENSG00000166033, ENSG00000169495
			PF00219	IGFBP	PF13365	Trypsin_2	ENSG00000166033, ENSG00000169495
			PF07648	Kazal_2	PF13365	Trypsin_2	ENSG00000166033, ENSG00000169495
VGG10	Hyaluronan and proteoglycan link protein 2	ENSG00000132702	PF00047	ig	PF00193	Xlink	ENSG00000140511, ENSG00000132692, ENSG00000187664
			PF00193	Xlink	PF07686	V‐set	ENSG00000157766, ENSG00000140511, ENSG00000132692, ENSG00000130287, ENSG00000187664, ENSG00000038427, ENSG00000145681
			PF00193	Xlink	PF13927	Ig_3	ENSG00000140511, ENSG00000187664, ENSG00000145681
VGG11	Integrin subunit beta 4	ENSG00000132470	PF03160	Calx‐beta	PF07965	Integrin_B_tail	
			PF07965	Integrin_B_tail	PF16656	Pur_ac_phosph_N	
VGG12	Mannose receptor C‐type 1	ENSG00000260314	PF00040	fn2	PF00652	Ricin_B_lectin	
			PF00652	Ricin_B_lectin	PF05473	UL45	
VGG13	MARVEL domain containing 2	ENSG00000152939	PF01284	MARVEL	PF07303	Occludin_ELL	ENSG00000274671, ENSG00000273814, ENSG00000197822
VGG14	Matrilin 4	ENSG00000124159	PF00008	EGF	PF10393	Matrilin_ccoil	ENSG00000132561, ENSG00000132031
			PF00092	VWA	PF10393	Matrilin_ccoil	ENSG00000162510, ENSG00000132561, ENSG00000132031
			PF07645	EGF_CA	PF10393	Matrilin_ccoil	ENSG00000162510, ENSG00000132561, ENSG00000132031
			PF10393	Matrilin_ccoil	PF12662	cEGF	ENSG00000162510, ENSG00000132561, ENSG00000132031
			PF10393	Matrilin_ccoil	PF13519	VWA_2	ENSG00000162510, ENSG00000132561, ENSG00000132031
			PF10393	Matrilin_ccoil	PF14670	FXa_inhibition	ENSG00000162510, ENSG00000132561, ENSG00000132031
VGG15	Matrix metallopeptidase 23B	ENSG00000189409	PF00413	Peptidase_M10	PF13895	Ig_2	
			PF00413	Peptidase_M10	PF13927	Ig_3	
VGG16	Matrix metallopeptidase 24	ENSG00000125966	PF00045	Hemopexin	PF11857	DUF3377	ENSG00000102996, ENSG00000157227, ENSG00000156103
			PF00413	Peptidase_M10	PF11857	DUF3377	ENSG00000102996, ENSG00000157227, ENSG00000156103
			PF01400	Astacin	PF11857	DUF3377	ENSG00000157227, ENSG00000156103
			PF01471	PG_binding_1	PF11857	DUF3377	ENSG00000102996, ENSG00000157227, ENSG00000156103
VGG17	Multimerin 1	ENSG00000138722	PF00386	C1q	PF07546	EMI	ENSG00000132205, ENSG00000138080, ENSG00000173269
VGG18	Platelet derived growth factor C	ENSG00000145431	PF00341	PDGF	PF00431	CUB	ENSG00000170962
VGG19	Protein C, inactivator of coagulation factors Va and VIIIa	ENSG00000115718	PF00089	Trypsin	PF00594	Gla	ENSG00000101981, ENSG00000057593, ENSG00000126231, ENSG00000126218, ENSG00000180210
			PF00594	Gla	PF09342	DUF1986	ENSG00000057593, ENSG00000101981
			PF00594	Gla	PF13365	Trypsin_2	ENSG00000101981, ENSG00000126218
VGG20	Protein tyrosine phosphatase, receptor type Z1	ENSG00000106278	PF00041	fn3	PF00194	Carb_anhydrase	ENSG00000144724
			PF00102	Y_phosphatase	PF00194	Carb_anhydrase	ENSG00000144724
			PF00194	Carb_anhydrase	PF00782	DSPc	ENSG00000144724
VGG21	Retinoid X receptor gamma	ENSG00000143171	PF00104	Hormone_recep	PF11825	Nuc_recep‐AF1	ENSG00000235712, ENSG00000228333, ENSG00000227322, ENSG00000231321, ENSG00000206289, ENSG00000186350, ENSG00000204231
			PF00105	zf‐C4	PF11825	Nuc_recep‐AF1	ENSG00000235712, ENSG00000228333, ENSG00000227322, ENSG00000231321, ENSG00000206289, ENSG00000186350, ENSG00000204231
VGG22	Retinol binding protein 3	ENSG00000265203	PF03572	Peptidase_S41	PF11918	Peptidase_S41_N	
VGG23	SATB homeobox 2	ENSG00000119042	PF02376	CUT	PF16534	ULD	ENSG00000182568
VGG24	SEL1L ERAD E3 ligase adaptor subunit	ENSG00000071537	PF00040	fn2	PF08238	Sel1	
VGG25	Semaphorin 4G	ENSG00000095539	PF01437	PSI	PF13895	Ig_2	ENSG00000138623, ENSG00000168758
VGG26	Sushi domain containing 2	ENSG00000099994	PF01033	Somatomedin_B	PF03782	AMOP	
VGG27	Synaptotagmin 2	ENSG00000143858	PF00168	C2	PF15807	MAP17	
VGG28	TNFRSF1A associated via death domain	ENSG00000102871	PF00531	Death	PF09034	TRADD_N	
VGG29	Transient receptor potential cation channel subfamily M member 7	ENSG00000092439	PF00520	Ion_trans	PF02816	Alpha_kinase	ENSG00000119121
			PF02816	Alpha_kinase	PF16519	TRPM_tetra	ENSG00000119121
VGG30	Tyrosine kinase non receptor 2	ENSG00000061938	PF07714	Pkinase_Tyr	PF11555	Inhibitor_Mig‐6	
			PF11555	Inhibitor_Mig‐6	PF14555	UBA_4	
VGG31	Zona pellucida glycoprotein 4	ENSG00000116996	PF00088	Trefoil	PF00100	Zona_pellucida	

Abbreviations: DSO‐Gs, domain‐shuffling origin genes; DSO‐GGs, domain‐shuffling origin gene groups; DSO‐DPs, domain‐shuffling origin domain pairs.

^a^ ENSEMBL gene IDs including the vertebrate DSO‐DPs under the threshold of E‐value < 1e−3.

**Table 2:** The list of the gnathostome DSO‐DPs and genes.

DSO‐GGs	Represented DSO‐Gs		DSO‐DPs				Other DSO‐Gs
(=Gene group ID)	Description	ENSEMBL gene ID	Domain 1		Domain 2		(=Gene IDs^a^)
GGG01	Butyrophilin subfamily 1 member A1	ENSG00000124557	PF00622	SPRY	PF08205	C2‐set_2	
			PF00047	ig	PF00622	SPRY	ENSG00000112763, ENSG00000124508, ENSG00000026950, ENSG00000111801, ENSG00000164010
			PF00622	SPRY	PF13927	Ig_3	ENSG00000113303, ENSG00000168903, ENSG00000165810, ENSG00000112763, ENSG00000124508, ENSG00000026950, ENSG00000111801, ENSG00000164010
GGG02	Myosin X	ENSG00000145555	PF00063	Myosin_head	PF16735	MYO10_CC	
			PF00169	PH	PF16735	MYO10_CC	
			PF00373	FERM_M	PF16735	MYO10_CC	
			PF00612	IQ	PF16735	MYO10_CC	
			PF00784	MyTH4	PF16735	MYO10_CC	
			PF00788	RA	PF16735	MYO10_CC	
			PF14593	PH_3	PF16735	MYO10_CC	
			PF15409	PH_8	PF16735	MYO10_CC	
			PF15413	PH_11	PF16735	MYO10_CC	
GGG03	OCRL, inositol polyphosphate‐5‐phosphatase	ENSG00000122126	PF00620	RhoGAP	PF16726	OCRL_clath_bd	
			PF03372	Exo_endo_phos	PF16726	OCRL_clath_bd	
GGG04	Sushi repeat containing protein, X‐linked 2	ENSG00000102359	PF00084	Sushi	PF13778	DUF4174	ENSG00000101955
			PF02494	HYR	PF13778	DUF4174	ENSG00000101955
GGG05	UEV and lactate/malate dehyrogenase domains	ENSG00000151116	PF00056	Ldh_1_N	PF05743	UEV	
			PF02866	Ldh_1_C	PF05743	UEV	
GGG06	Zinc finger protein 536	ENSG00000198597	PF00096	zf‐C2H2	PF16606	zf‐C2H2_assoc	
			PF05605	zf‐Di19	PF16606	zf‐C2H2_assoc	
			PF13465	zf‐H2C2_2	PF16606	zf‐C2H2_assoc	
			PF13894	zf‐C2H2_4	PF16606	zf‐C2H2_assoc	
			PF13909	zf‐H2C2_5	PF16606	zf‐C2H2_assoc	

Abbreviations: DSO‐Gs, domain‐shuffling origin genes; DSO‐GGs, domain‐shuffling origin gene groups; DSO‐DPs, domain‐shuffling origin domain pairs.

^a^ ENSEMBL gene IDs including the gnathostome DSO‐DPs under the threshold of E‐value < 1e−3.

**Table 3:** The list of the cyclostome DSO‐DPs and genes.

DSO‐GGs	Represented DSO‐Gs		DSO‐DPs				Other DSO‐Gs
(=Gene group ID)	Description^a^	Gene ID^b^	Domain 1		Domain 2		(=Gene IDs^c^)
CGG01	ARL8B_homolog	g6788	PF00025	Arf	PF00583	Acetyltransf_1	
			PF00503	G‐alpha	PF00583	Acetyltransf_1	
			PF00583	Acetyltransf_1	PF01926	MMR_HSR1	
			PF00583	Acetyltransf_1	PF04670	Gtr1_RagA	
			PF00583	Acetyltransf_1	PF08477	Roc	
			PF00583	Acetyltransf_1	PF09439	SRPRB	
CGG02	CCDC94_homolog	g2903	PF04502	DUF572	PF14936	p53‐inducible11	
CGG03	DOCK1_homolog	g2681	PF08506	Cse1	PF14429	DOCK‐C2	
CGG04	HYI_homolog	g1738	PF01261	AP_endonuc_2	PF01907	Ribosomal_L37e	
CGG05	KCNS3_homolog	g4934	PF02724	CDC45	PF10446	DUF2457	
CGG06	MCAM_homolog	g10860	PF00047	ig	PF12301	CD99L2	
			PF07679	I‐set	PF12301	CD99L2	
			PF07686	V‐set	PF12301	CD99L2	
			PF08205	C2‐set_2	PF12301	CD99L2	
			PF12301	CD99L2	PF13895	Ig_2	
			PF12301	CD99L2	PF13927	Ig_3	
CGG07	NWD2_homolog	g4096	PF07569	Hira	PF13271	DUF4062	
CGG08	PARG_homolog	g7740	PF05028	PARG_cat	PF05716	AKAP_110	
CGG09	PPAT_homolog	g4078	PF00153	Mito_carr	PF00156	Pribosyltran	
CGG10	PROX1_homolog	g2781	PF00769	ERM	PF05044	HPD	
CGG11	SETMAR_homolog	g18133	PF13565	HTH_32	PF16087	DUF4817	
CGG12	SLIT2_homolog	g14957	PF11921	DUF3439	PF12799	LRR_4	g24021
			PF11921	DUF3439	PF13306	LRR_5	
			PF11921	DUF3439	PF13855	LRR_8	g24021
CGG13	TIGD1_homolog	g15228	PF01722	BolA	PF04218	CENP‐B_N	
			PF04218	CENP‐B_N	PF13465	zf‐H2C2_2	g17584
			PF04218	CENP‐B_N	PF13894	zf‐C2H2_4	g5746, g17584
CGG14	TIGD2_homolog	g5746	PF13518	HTH_28	PF13894	zf‐C2H2_4	
CGG15	TOM1L2_homolog	g7950	PF00790	VHS	PF15002	ERK‐JNK_inhib	
			PF03127	GAT	PF15002	ERK‐JNK_inhib	
CGG16	TRIM14_homolog	g1602	PF00020	TNFR_c6	PF00622	SPRY	g14212
			PF00020	TNFR_c6	PF13765	PRY	g14212, g14213
CGG17	zinc finger protein 3‐like isoform X1	LOC116946555	PF13465	zf‐H2C2_2	PF13518	HTH_28	

Abbreviations: DSO‐Gs, domain‐shuffling origin genes; DSO‐GGs, domain‐shuffling origin gene groups; DSO‐DPs, domain‐shuffling origin domain pairs.

^a^ Gene name in the GRAS‐LJ or Refseq.

^b^ Gene IDs assigned in the GRAS‐LJ or Refseq.

^c^ GRAS gene IDs including the cyclostome DSO‐DPs under the threshold of E‐value < 1e−3.

We noted that the gene groups may not be composed of orthologous or paralogous genes, because we did not consider the sequence similarity of whole amino acid sequences and domain architectures beyond focal DSO‐DPs (e.g., VGG10 lecticans are included in the same gene groups as hyaluronan proteoglycan link proteins). We also did not consider the convergent acquisition of the same DSO‐DP.

### 2.6 Annotation of Lamprey DSO Genes

To annotate DSO genes, we conducted a BLAST search of the amino acid sequences of lamprey DSO genes against the Swiss‐Prot database (Bateman et al. 2021). We set the threshold as an E‐value lower than 1e−10 and extracted the best hit of each gene.

### 2.7 Gene Ontology (GO) Analysis

GO was used to infer the potential functions of DSO‐Gs (Ashburner et al. 2000). We performed GO enrichment analysis for human DSO‐Gs on PANTHER (Mi et al. 2019) using Fisher's exact test, and the false discovery rate (FDR) was used to filter for significantly enriched GO terms (FDR < 0.05).

For the functional annotation of each gene group, we merged GO annotations for the DSO‐Gs within the corresponding DSO‐GGs and then counted the annotated GO terms. We used the GO annotations included in the results of the GO enrichment analysis. We created figures using the “tidyverse” and “ggplot2” packages in R version 4.3.2 (R Center For Statistical Computing, Vienna, Austria).

### 2.8 Sample Collection of Arctic Lampreys

Adult Arctic lampreys (*L*. *camtschaticum*) were collected from Shiribeshi‐Toshibetsu River, Hokkaido, Japan. For artificial fertilization, we anesthetized the lampreys using 0.20 g/L ethyl 3‐aminobenzoate methanesulfonate (MS‐222, Sigma‐Aldrich, St. Louis, MO, USA) and collected mature eggs and sperm. The fertilized eggs were cultured at 16°C. We identified lamprey embryonic stages as previously described (Tahara 1988).

### 2.9 In Situ Hybridization of Arctic Lamprey

Whole‐mount in situ hybridization of stage 29 Arctic lamprey embryos was performed as previously described, with minor modifications (Ogasawara et al. 2000). The cDNA library of Arctic lamprey was prepared by extracting RNA from stage 26 and stage 28 embryos using TRIzol reagent (Life Technologies, Carlsbad, CA, USA) and TRIzol/chloroform extraction following the manufacturer's instructions and performing reverse transcription with PrimeScript 1st strand cDNA Synthesis Kit (Takara Bio, Shiga, Japan). We used the primers listed in Supporting Information S2: Table S5 (note that reverse primers with a 20‐bp T3 promoter sequence was used) to synthesize probes from the cDNA library by nested polymerase chain reaction (PCR) using PrimeSTAR GXL DNA Polymerase kits (Takara Bio, Shiga, Japan). We used some of the embryos for preparing frozen sections with thicknesses of 10–20 μm.

### 2.10 Sample Collection of Brook Lamprey

Brook lamprey (*L*. sp southern) larvae were collected from the lower reaches of Wada River in Toyama, Japan. Lamprey metamorphosis stages were identified as previously described (Youson and Potter 1979). Brook lampreys were cultured at 16°C in the dark. Finally, we used a larva of approximately 120 mm in length, three late‐metamorphosis stage larvae, and an adult for RNA extraction.

### 2.11 RNA Sequencing of Brook Lamprey

To extract total RNA from the whole bodies of the five brook lampreys, we anesthetized the lampreys, dissected them on ice using post‐mortem scissors, and homogenized the whole bodies in TRIzol reagent (Life Technologies). RNA was extracted using TRIzol/chloroform following the manufacturer's instructions. Genomic DNA was digested from RNA samples using RNeasy kit (Qiagen, Hilden, Germany). RNA sequencing was conducted using a NovaSeq. 6000 (Illumina Inc., San Diego, CA, USA), with 100‐bp paired‐end sequencing. Library preparation and sequencing were performed by Macrogen Inc. (Seoul, South Korea).

We removed adaptors and low‐quality sequences using Trimmomatic version 0.38 (Bolger, Lohse, and Usadel 2014) and FastQC version 0.11.9 (Andrews 2010) to evaluate the quality of the sequence data.

### 2.12 Transcriptome Assembly of Brook Lamprey RN A‐Seq Data

After quality control of the RNA‐seq data of brook lamprey, we performed de novo transcriptome assembly using Trinity version 2.8.4 (Grabherr et al. 2011) with the parameters “‐‐seqType fq ‐‐max_memory 80G ‐‐SS_lib_type FR” on the RNA‐seq data from the three stages altogether.

### 2.13 Transcriptome Assembly of Arctic Lamprey RNA‐Seq Data

For lamprey embryonic stage data, we used published RNA‐seq data of eight stages of Arctic lamprey embryos from stages 12, 16, 18, 20, 22, 24, 26, and 28 (Pascual‐Anaya et al. 2018) and conducted de novo transcriptome assembly using Trinity.

### 2.14 Validation of Lamprey Gene Models Using RNA‐Seq Data

We used the assembled transcripts from lamprey RNA‐seq data sets to assess the expression of DSO‐Gs in lamprey. First, we predicted protein sequences from the lamprey‐assembled transcripts using Transdecoder version 5.5.0 (Haas et al. 2013). To identify sequences corresponding to the gene models, we conducted a BLAST search of the amino acid sequences of the assembled transcripts against gene models. We set the threshold as an E‐value lower than 1e‐50 and an identity higher than 90%.

We used HMMER3 and Pfam‐A for the domain search as described above. Domain pair enumeration was applied to the peptide sequences predicted using RNA‐seq. We then searched for DSO‐DPs in vertebrates and cyclostomes.

### 2.15 Phylogenetic Analysis of the Lamprey PTPRG‐Like Genes

For the phylogenetic analysis, we prepared homologous sequences of protein tyrosine phosphatase receptor type Z1 (PTPRZ1, also known as pleiotrophin receptor 1) using aLeaves (Kuraku et al. 2013). (Supporting Information S2: Table S6). We used full‐length sequences of three PTPRG‐like genes of *P*. *marinus* as the query. We reduced highly similar sequences using CD‐HIT version 4.8.1 (Fu et al. 2012) with the parameter, ‐c 0.95 and ‐M 4000. We identified the locations of the phosphatase domains using HMMER search described above and used the sequences of the two phosphatase domains for the phylogenetic analysis. For LcPTPRGLa, the phosphatase domains were truncated. Based on the alignment between LcPTPRGLa and homologous sequences in the brook lamprey RNA‐seq data, we identified phosphatase domains in adjacent gene models (g8341.t1). Therefore, we selected this sequence as the phosphatase domain for LcPTPRGLa. The sequences were aligned using MAFFT version 7.481 (Katoh and Standley 2013) and trimmed into poorly aligned regions using TrimAL version 1.4. rev.15, (Capella‐Gutiérrez, Silla‐Martínez, and Gabaldón 2009) with the “automated1” option. Phylogenetic analysis was conducted using RAxML version 8.2.12 (Stamatakis 2014) with the parameters “‐f a ‐x 12345 ‐p 12345 ‐# 500 ‐m PROTGAMMAAUTO ‐‐auto‐prot=aic ‐T 16” using the trimmed alignment.

### 2.16 Ethics Declarations

All procedures in this study were performed in compliance with the guidelines for animal use of the Animal Care Committees at University of Tsukuba (specific approval is not needed for experimentation on fishes under the Japanese law, Act on Welfare and Management of Animals). During the investigation, every effort was made to minimize suffering and to reduce the number of animals used.

## 3 Results

### 3.1 Candidates of the Novel Genes Acquired via Domain Shuffling in the Vertebrate Common Ancestor

To identify vertebrate domain‐shuffling origin genes (DSO‐Gs), we performed a comparative analysis of genes from 22 metazoan genomes (see Section 2.2). Assuming that genes contributed to the common characteristics of vertebrates are conserved among vertebrates, we searched for domain pairs conserved among all gnathostomes and at least one cyclostome species. Consequently, we identified 63 domain pairs as vertebrate DSO‐DPs, corresponding to 74 DSO‐Gs in the human genome (Table 1).

**Figure 2:**
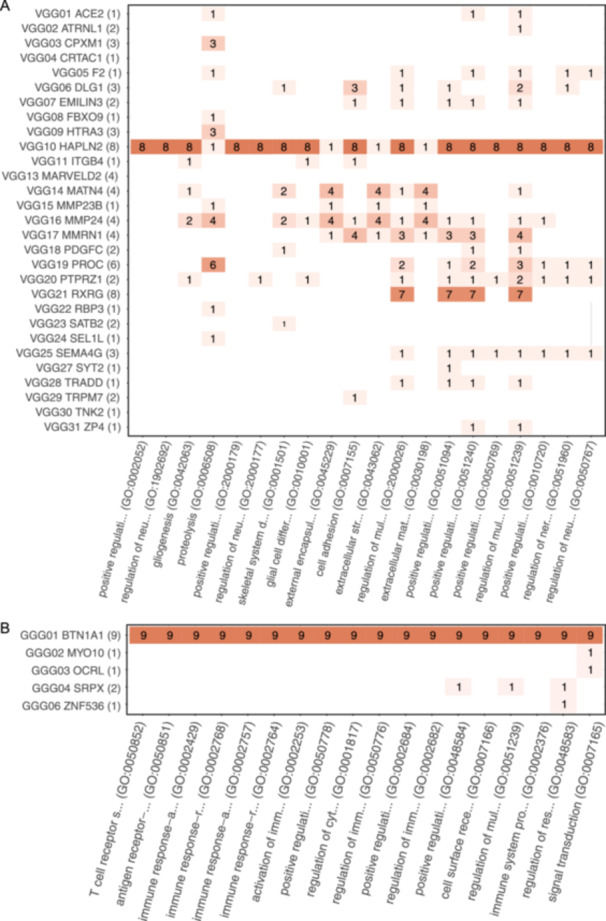
Enriched gene ontology (GO) terms in the vertebrate domain‐shuffling origin genes (DSO‐Gs) and the gnathostome DSO‐Gs. (A and B) The enriched GO terms in the vertebrate DSO‐Gs (A) and gnathostome DSO‐Gs (B). The x‐axis indicates the top 20 GO terms for vertebrate DSO‐Gs and the top 18 GO terms for gnathostome DSO‐Gs (false discovery rate [FDR] < 0.05). The y‐axis indicates the represented gene symbol of the gene groups. The tiles show the number of DSO‐Gs included in the DSO‐gene groups (DSO‐GG) and the number of DSO‐Gs annotated to the GO term. The color of each tile indicates the number of DSO‐Gs.

We inferred the potential functions of DSO‐Gs through GO enrichment analysis of the domain terms of biological processes to elucidate whether the identified vertebrate DSO‐Gs contributed to the evolution of novel vertebrate characteristics, including blood coagulation systems and head structures derived from neural crest cells and placodes. We used GO terms in the biological process domain to annotate the vertebrate DSO‐Gs. Using GO terms in the “biological process” domain, we identified 76 enriched GO terms for the vertebrate DSO‐Gs (FDR < 0.05; Supporting Information S2: Table S7), including “gliogenesis” (GO:0042063), “skeletal system formation” (GO:0001501) (Figure 2A), and “blood coagulation” (GO:0007596). These biological functions were associated with novel vertebrate characteristics.

To evaluate the influence of independent domain‐shuffling events on novel vertebrate characteristics, we performed GO term analysis for the vertebrate DSO‐GGs (genes sharing vertebrate DSO‐DPs) which would include genes acquired by the same domain‐shuffling event. We merged and counted GO terms per gene group (see Section 2.7). As a result, we found the annotation of “gliogenesis” from five DSO‐GGs, “skeletal system development” from six DSO‐GGs, and “blood coagulation” from three DSO‐GGs (Figure 2A and Supporting Information S2: Table S7). This suggests that multiple independent domain‐shuffling events have contributed to the emergence of novel traits during the early evolution of the vertebrates.

### 3.2 Vertebrate DSO‐Gs in Cyclostomes

To explore the evolutionary significance of vertebrate DSO‐Gs in the acquisition of novel characteristics in the common ancestor of the vertebrates, we examined whether vertebrate DSO‐Gs in the cyclostomes showed conserved functions with the gnathostomes. We detected 24 vertebrate DSO‐GGs composed of 44 vertebrate DSO‐Gs from the Arctic lamprey genome. These vertebrate DSO‐Gs comprised 48 of 63 vertebrate DSO‐DPs (Supporting Information S2: Table S8). Some of these vertebrate DSO‐GGs have been reported to be expressed in lampreys and have shared functions with gnathostomes and lampreys. For example, lectican genes (VGG10) are expressed in the cartilage and neural tube of lampreys, suggesting a conserved function as matrix proteins of the cartilage and nervous system in gnathostomes (Root et al. 2021). Homologous genes of occludin (VGG13), a protein associated with tight junctions in gnathostomes, are expressed in the skin, gills, and kidneys of lampreys and contribute to osmoregulation (Kolosov et al. 2017). In addition, coagulation factor VII (VGG19) contributes to blood coagulation (Beeler, Aird, and Grant 2018).

As a prerequisite for gene function, we examined whether vertebrate DSO‐Gs are expressed in lampreys. To this end, we conducted transcriptome assembly on the newly performed RNA‐seq data of whole‐mount brook lamprey at the larval, late‐metamorphosis, and adult stages and the published lamprey RNA‐seq data of lamprey embryos, resulting in the assembly of transcripts from the embryonic stage and the late larva to the adult stage. From the assembled transcript sets, we confirmed the expression of 21 (of 24) DSO‐GGs, composed 40 (of 44) DSO‐Gs (Supporting Information S2: Table S9). Moreover, we confirmed that the domain pairs of 44 of the 63 vertebrate DSO‐DPs colocalized on the same contigs in the lamprey transcriptome assembly data (Supporting Information S2: Table S10). Collectively, these results indicated that most vertebrate DSO‐Gs are transcribed during lamprey ontogeny and may play a role in lamprey development.

### 3.3 PTPRZ1, a Vertebrate DSO‐GG, Is Relevant to Myelination in Gnathostome

**Figure 3:**
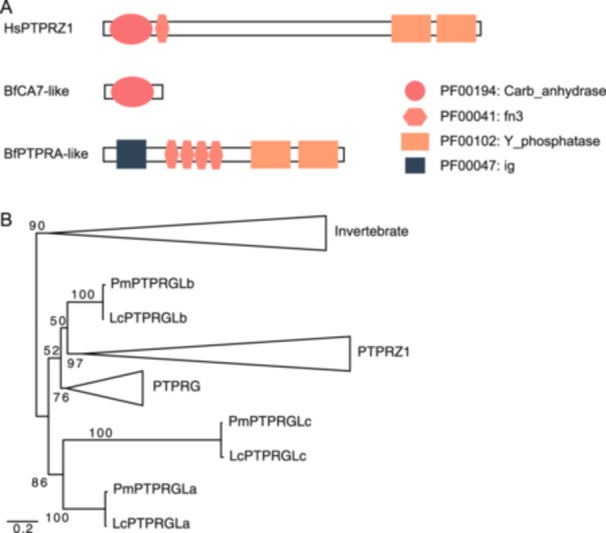
The structure of PTPRZ1 and the phylogeny of lamprey *PTPRG‐like* genes. (A) Domain architecture of PTPRG/PTPRZ1. HsPTPRZ1 architecture is based on ENSP00000377047, BfCA7‐like architecture is based on estExt_fgenesh2_pg.C_2610037, BfPTPRA‐like architecture is based on estExt_fgenesh2_pg.C_370011. (B) A molecular phylogeny of PTPRG/PTPRZ1 in vertebrates. The numbers of each branch indicate the bootstrap values. Lc, *Lethenteron camtschaticum*; Pm, *Petromyzon marinus*.

Among the vertebrate DSO‐Gs, attractin (VGG02, ATRN), disc large homolog 1 (VGG06, DLG1), and protein tyrosine phosphatase receptor type Z1 (VGG20, PTPRZ1) are associated with myelination, a novel trait of gnathostomes. Notably, PTPRZ1, which contributes to myelin sheath formation in the gnathostome (Harroch et al. 2000; Lamprianou et al. 2011), showed a drastic change in the domain architecture from its original genes in the common ancestor of vertebrates (Figure 3A).

Despite cyclostomes not having myelin sheaths, we found three homologous genes from the Arctic lamprey genome. These genes share the same DSO‐DPs with the gnathostome PTPRZ1 and sequence similarity search results showed significant similarity with gnathostome PTPRZ1 or PTPRG (Supporting Information S2: Table S11, E‐value < 1e−50). Thus, the gene groups should have appeared in the common ancestor of vertebrates. The function of these genes should contribute to the understanding of myeline evolution in early vertebrates. However, the functions of these genes in cyclostomes remain unknown. To explore the potential functions of these genes in cyclostomes, we performed phylogenetic and expression analyses of the *PTPRZ1* homologs in lamprey.

We conducted a phylogenetic analysis to understand the relationship between these lamprey genes and gnathostome PTPRG/PTPRZ1. However, we could not reconstruct a phylogenetic tree with statistical support (Figure 3B). Therefore, we could not determine the relationship between lamprey PTPRZ1 homologs and gnathostome PTPRG/PTPRZ1. The hagfish homolog of PTPRG‐like is suggested as an orthologue of PTPRG based on the C terminal domain sequences analysis (Ono‐Koyanagi et al. 2000). Therefore, we named the three lamprey PTPRG‐like genes as *LcPTPRGLa*, *LcPTPRGLb*, and *LcPTPRGLc*.

**Figure 4:**
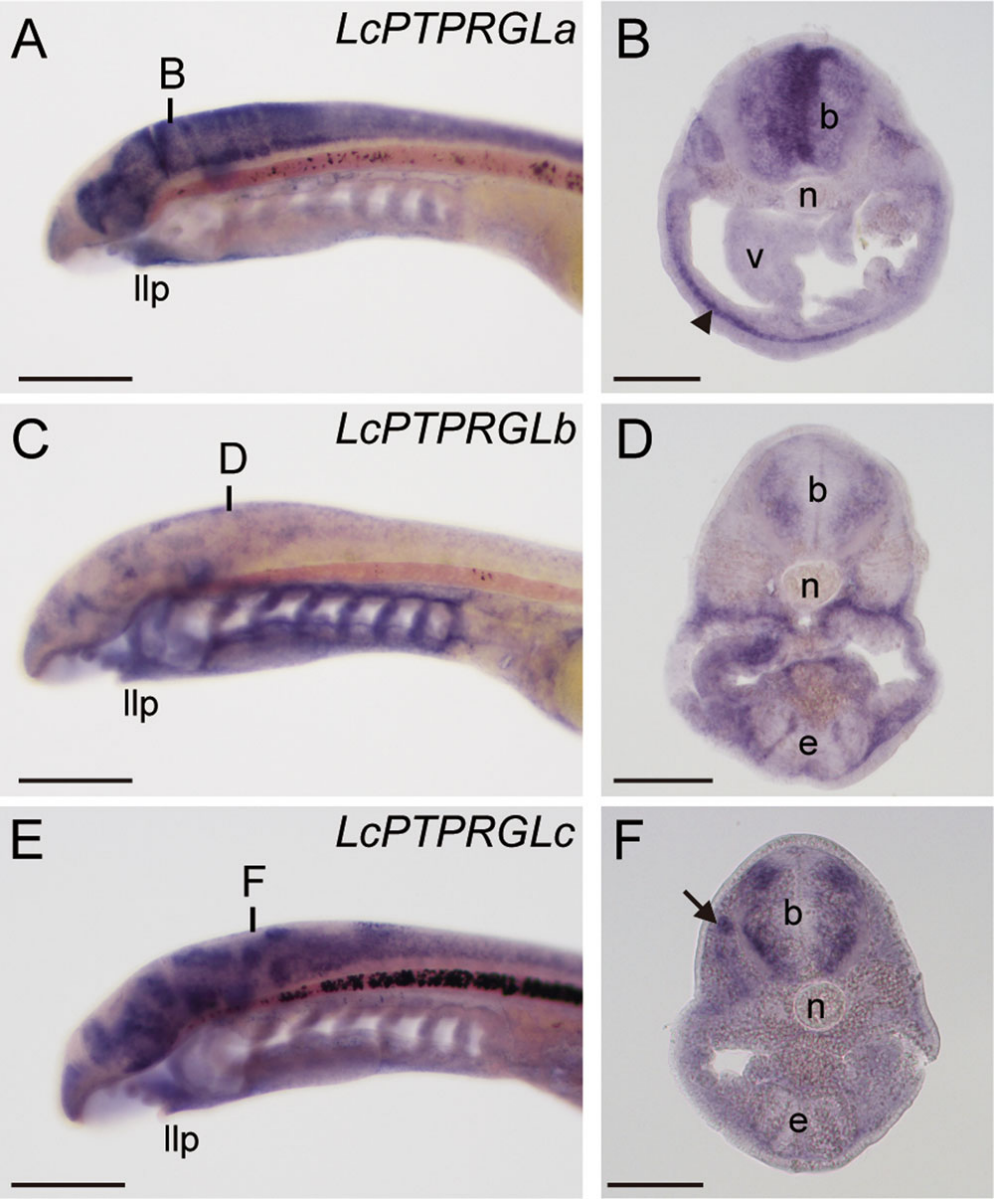
The expression of lamprey *PTPRG‐like* genes. (A, C, E) Whole mount in situ hybridization of stage 29 embryos for the expression of *LcPTPRGLa* (A), *LcPTPRGLb* (C), and *LcPTPRGLc* (E). Vertical lines indicate the approximate positions of transverse sections in B, D, and F. Scale bars: 200 μm. llp, lower lip. (B, D, F) Transverse sections of stage 29 embryos of *LcPTPRGLa* (B), *LcPTPRGLb* (D), and *LcPTPRGLc* (F). The black arrow shows expression in a sensor ganglion. The black arrowhead shows expression in muco‐cartilage. Scale bars: 100 μm. b, brain. e, endostyle. n, notochord. v, velum.

Next, we analyzed the spatial expression patterns of these genes in the lamprey embryos. At stage 29, *LcPTPRGLa* expression was observed in the brain and the lower lip mucocartilage which is a unique lamprey tissue derived from the neural crest cells (Figure 4A,B). *LcPTPRGLb* was expressed in the brain and the mesenchyme in the branchial region (Figure 4C,D). In contrast, *LcPTPRGLc* expression was observed in the brain and the sensory ganglia (Figure 4E,F). These results indicate that the expression of *PTPRG/PTPRZ1* is conserved in the brain and the sensory ganglia, suggesting that *PTPRG/PTPRZ1* functions in neural crest cells or placodes in the common ancestor of vertebrates.

Additionally, we investigated the cell‐type‐specific expression of *PTPRG‐like* genes using the brain atlas data of the sea lampreys (Lamanna et al. 2023, https://downloads.kaessmannlab.org/lamprey/). In the adult sea lampreys, the expression of *PmPTPRGLb* and *PmPTPRGLc* was detected in only a subset of neurons, astrocytes, fibroblasts, and vascular cells, while *PmPTPRGLa* showed high expression in astrocytes (Supporting Information S5: Figure S3). In contrast, in the larval stage of the sea lampreys, the expression of *PmPTPRGLb* and *PmPTPRGLc* was weakly detected in neurons and astrocytes. In summary, the tissue‐level and the cell‐type‐level expression patterns in the lampreys suggest that the *PTPRG‐like* genes are associated with the development and evolution of glial cells.

### 3.4 Candidates for Novel Gnathostome DSO‐Gs Include Genes Relevant to Novel Characteristics of Gnathostomes

Next, we focused on the DSO‐Gs acquired in the common ancestor of gnathostomes. Searching for domain pairs conserved among all gnathostomes, we identified 23 domain pairs as vertebrate DSO‐DPs, corresponding to 15 DSO‐Gs in the human genome (Table 2). The common ancestor of gnathostomes acquired novel characteristics such as jaw morphology, paired appendages, myelinated nerves, and unique adaptive immune systems. If the gnathostome DSO‐Gs were associated with these characteristics, we considered them to have contributed to the emergence of novel traits in gnathostomes. We conducted a GO analysis of the human gnathostome DSO‐Gs using GO terms in the biological process domain (Figure 2B). The GO enrichment analysis identified 18 enriched GO terms (FDR < 0.05; Supporting Information S2: Table S12 and Figure 2B). We detected GO terms associated with immune responses such as “T cell receptor signaling pathway” (GO:0050852) and “regulation of immune system process” (GO:0002682) (Supporting Information S2: Table S12). Because the adaptive immune systems of gnathostomes differ from those of cyclostomes (Rast and Buckley 2013), parts of the gnathostome DSO‐Gs are associated with the immune system which is a novel character of gnathostomes.

A gnathostome DSO‐G was associated with myelination, although these characteristics were not enriched in the GO analysis. Zinc finger protein 536 (GGG06, ZNF536) is associated with oligodendrocyte differentiation (Dugas et al. 2006). ZNF536 is annotated to “regulation of gene expression” (GO:0010468). This indicates that novel domain architectures acquired via domain shuffling may contribute to the evolution of myelin by regulating gene expression.

### 3.5 Candidates for the Novel Gene of Cyclostomes

Finally, we extracted DSO‐Gs acquired from the common ancestor of the cyclostomes. Searching for domain pairs conserved between at least one of the hagfishes and the lampreys, we identified 33 domain pairs as cyclostome DSO‐DPs, corresponding to 20 DSO‐Gs in the Arctic lamprey genome (Table 3). To our knowledge, these genes were not annotated to any function. Thus, we inferred their function from the domains included in the genes.

Among the 17 cyclostome DSO‐GGs, domain annotations suggested CGG16 (TRIM14_homolog) might be involved in immunity. This gene group has a novel domain pair composed of “TNFR_c6” and “PRY.” TNFR_c6 is a binding domain to tumor necrosis factor, an important signal of the immune system (Banner et al. 1993). PRY is found in Tripartite motif protein family genes which function in antiviral response and are coupled with E3 ubiquitin ligase activity (D'Cruz et al. 2013). Since both domains are located within the genes associated with the immune system, CGG16 may be associated with the cyclostome‐type immune system.

We examined whether the cyclostome DSO‐Gs were expressed using the lamprey transcriptome data. We identified homologous genes in the Arctic lamprey and brook lamprey transcriptome data for 16 of the cyclostome DSO‐GGs (comprising 32 domain pairs) (Supporting Information S2: Table S13). Among the 17 DSO‐GGs, the 18 cyclostome DSO‐DP was located on the same transcript in the lamprey transcriptome data (Supporting Information S2: Table S14). These results suggest that the cyclostome DSO‐Gs are at least transcribed in lampreys.

## 4 Discussion

### 4.1 Candidate Genes With Novel Domain Architectures Acquired in the Common Ancestor of Vertebrates, Gnathostomes, and Cyclostomes

In this study, we utilized the recent availability of the cyclostome genomes to analyze the genomes of 22 metazoans and detected candidate genes acquired via domain shuffling in common ancestors of vertebrates, gnathostomes, and cyclostomes. We identified 74 human genes as vertebrate DSO‐Gs (31 DSO‐GGs), 15 human genes as gnathostome DSO‐Gs (6 DSO‐GGs), and 20 Arctic lamprey genes as cyclostome DSO‐Gs (16 DSO‐GGs of 17 cyclostome DSO‐GGs) (Tables 1, 2, 3). The identification of these DSO‐Gs allowed us to compare the acquisition timings of novel domain architectures and novel characteristics before and after the divergence of gnathostomes and cyclostomes. Based on our analyses, we suggest that DSO‐Gs that have novel domain architectures acquired via domain shuffling contributed to the evolution of each clade, as discussed below.

To search for DSO‐Gs, we used domain pairs that have been suggested as evolutionary markers of novel characteristics (Chothia et al. 2003). The domain‐pair approach allows the comparison of domain architectures that are hardly detected by local alignment (e.g., BLAST). Furthermore, this approach is advantageous in detecting DSO‐Gs if gene models from draft genomes include misassembled genes. Our domain pair analysis detected many novel gene candidates that appear to have resulted from domain‐shuffling events in the common ancestors of vertebrates, gnathostomes, and cyclostomes.

Based on GO analysis, DSO‐Gs were associated with novel characteristics of vertebrate or gnathostome lineages. Among the vertebrate DSO‐Gs, seven genes were annotated to blood coagulation, 15 genes were annotated to skeletal system development, 13 genes were annotated to gliogenesis, and 12 genes were annotated to regulation of nervous system development (Figure 2A). The vertebrate blood coagulation system is partially conserved between cyclostomes and gnathostomes but differs from that of invertebrates (Beeler, Aird, and Grant 2018; Jiang and Doolittle 2003; Loof et al. 2011). Consistent with our results, previous studies suggested that vertebrate blood coagulation system occurs via domain shuffling in the deuterostome lineage (Coban, Bornberg‐Bauer, and Kemena 2022; Kawashima et al. 2009; Patthy 2003). Regarding the vertebrate DSO‐Gs associated with skeletal system development, our results included aggrecan (VGG10) and SATB2 (VGG23). SATB2 is a transcription factor that encompasses a homeodomain, is expressed in the pharyngeal arch, and is associated with craniofacial patterning (Dobreva et al. 2006; Sheehan‐Rooney et al. 2010). A previous study suggested that *SATB* genes were acquired via domain shuffling based on a comparison between the protochordate and euteleostomi (Takatori and Saiga 2008). Our analysis increased the resolution of the acquisition timing of *SATB* genes. During nervous system development, *PTPRG* is expressed in the sensory ganglia or sensory organs (Lamprianou et al. 2006) which are included in the cranial structure, a novel characteristic of vertebrates. These results suggested that the identified vertebrate DSO‐Gs were acquired via domain shuffling during the evolution of early vertebrates and are associated with novel vertebrate characteristics.

Among the gnathostome DSO‐Gs, the genes of butyrophilin subfamily (GGG01) are included in the gnathostome DSO‐Gs, and they have signaling and antigen presentation associated with the γδ T cells (Arnett and Viney 2014). Based on the expression of transcription factors and signaling molecules, the cyclostomes have homologous cells to αβ T cells, γδ T cells, and B cells of gnathostomes (Hirano et al. 2013). However, the antigen‐recognizing receptor and antigen presentation systems of cyclostomes are different from those of gnathostomes (Das et al. 2015; Rast and Buckley 2013). These genes may be associated with the evolution of the novel immune system characteristics in gnathostomes.

Among the identified cyclostome DSO‐Gs, three genes of Arctic lampreys (CGG16, TRIM14_homolog) may be associated with immunity. The immune system of cyclostomes differs from that of gnathostomes. Thus, domain shuffling may have contributed to the evolution of cyclostome characteristics such as in gnathostomes and vertebrates, although further investigations on the function are required. The analysis of the DSO‐Gs may contribute to understanding the unique characteristics of the adaptive immune system in cyclostomes.

Collectively, our results indicated that the timing of the acquisition of the novel domain architectures via domain shuffling was mostly consistent with the emergence of the novel characteristics in the evolution of vertebrates, gnathostomes, and cyclostomes.

### 4.2 Inconsistency Between Gene Acquisition and Characteristics Emergence

The myelin sheath is a novel trait in gnathostomes that contributes to nerve protection, the rapid transmission of electric impulses, and neuronal metabolic maintenance (Zalc 2016). In this study, while a gnathostome DSO‐G was related to myelination, three vertebrate DSO‐Gs (PTPRZ1, ATRN, and DLG1) were also associated with signaling and vesicle trafficking in myelination (Bolis et al. 2009; Kuramoto et al. 2001; Lamprianou et al. 2011). Thus, the acquisition of some of the myelination‐related DSO‐Gs precedes the emergence of gnathostome‐type myelination. Consistently, previous studies have reported that some myelination‐associated genes are conserved in sea lamprey and ascidian genomes (Gould et al. 2005; Smith et al. 2013), and the acquisition of several other myelination‐associated genes occurred in the common ancestor of gnathostomes (Werner 2013). Thus, our results suggest that the myelination‐associated vertebrate DSO‐Gs were newly recruited for myelination in the common ancestor of gnathostomes.

The preceding acquisition of *PTPRG/PTPRZ1* to myelination was supported by the gene expression patterns. We showed that the *LcPTPRGL* genes, which are homologous to *PTPRZ1* in Arctic lampreys, are expressed in the brain, sensory ganglions, and lower lip (Figure 4). *PTPRG*/*PTPRZ1* in gnathostomes are also expressed in various cell types in the nervous system, including oligodendrocytes, neurons, astrocytes, and microglia (Lamprianou et al. 2006; Shintani et al. 1998). These results suggest that *PTPRG/PTPRZ1* is associated with the central nervous system and derivatives of neural crest cells in a common ancestor of vertebrates. Therefore, the inconsistency of *PTPRZ1* acquisition is also explained by recruitment.

Investigating the function of myelin‐associated genes in cyclostomes should contribute to our understanding of the evolution of myelination. Because a large part of lamprey glial cells in the central nervous system are assumed to be astrocytes (Rovainen 1982), the homology between the glial cells of lampreys and gnathostomes remains unclear (Hines 2021). Some studies have suggested that oligodendrocytes originate from motor glial cells (Richardson et al. 1997) while others have suggested that they originate from metabolic interactions between neurons and glial cells (Nave, Tzvetanova, and Schirmeier 2017). Both ideas are supported by morphological and genetic studies on lamprey (Weil et al. 2018; Yuan, York, and McCauley 2018). The lamprey brain atlas data show sea lamprey *PTPRG‐like* genes are expressed in astrocytes, supporting that *PTPRG/PTPRZ1* genes might already have any function in astrocytes of the common ancestor of vertebrates. In contrast, other myelination‐associated genes, are not expressed in astrocytes but in fibroblasts (Lamanna et al. 2023). Thus, additional studies are required to identify signaling pathways and regulation of *PTPRG‐like* genes in lamprey, to infer the ancestral states of myelin‐associated glial cells.

### 4.3 Potential Limitations and Future Directions

This study had several limitations and future directions. First, a potential limitation of this study is the lower number of detected domains in cyclostomes, particularly hagfishes, compared to jawed vertebrates. While our analysis revealed a significant difference in the number of detected domains between cyclostomes and gnathostomes, this discrepancy could not be explained by GC content or transcript abundance by several statistical tests (data not shown). These findings suggest that lineage‐specific genomic features may contribute to the variation in domain detection in cyclostomes. Future work should refine detection methods and explore other genomic factors, while the availability of higher‐quality genome sequences and gene models may also improve domain identification across lineages.

Second, although we found DSO‐Gs to be associated with the novel characteristics, it is unclear whether the acquisition of new domain pairs itself contributes to the novel characteristics. For example, it is plausible that the acquisition of phenotypic traits may be sufficiently mediated by a single domain of a certain DSO‐G, rather than by domain pairs. Follow‐up functional analyses are required to investigate the significance of domain shuffling in novel traits.

Third, our analysis focused on the pairing of domains and not the order of the domain pairs in the genes. Previous studies suggest that nonrandom chromosome rearrangements may constrain the order of the domain pairs (Murphy et al. 2005; Pevzner and Tesler 2003). Consistently, when we considered the order of the domains in the domain‐pair search, the results were mostly unchanged (data not shown). However, considering the order of the domains in the genes might provide additional information if the acquisition of a certain domain pair is rather frequent in the evolution of vertebrates.

Fourth, our domain‐pair search may have missed some potential DSO‐DPs. In this study, based on the assumption of “one acquisition and multiple losses,” we searched for highly conserved domain pairs to detect DSO‐Gs associated with novel characters. This procedure may have overlooked DSO‐DPs acquired through convergence. Although the convergent evolution of domain pairs is estimated to be a rare event (Gough 2005), some researchers have reported that convergent DSO‐G evolution occurs in immune receptor genes among metazoan lineages (Zhang, Zmasek, and Godzik 2010). Currently, the genomes of numerous species are available (Lewin et al. 2018) and with more genomic data from various lineages, we can possibly create a better estimation model for gene origin and secondary gene loss. These comparative analyses help identify novel domain architectures that lead to the acquisition of novel phylogenetic traits.

## 5 Conclusions

In summary, we identified genes with novel domain architectures acquired via domain shuffling during vertebrate evolution and successfully determined whether their acquisition occurred before or after the diversification of gnathostomes and cyclostomes. We found that many of the DSO‐Gs acquired in the common ancestor of vertebrates, gnathostomes, and cyclostomes were associated with novel characteristics of the respective lineages. Notably, our results showed that some vertebrate DSO‐Gs were associated with a novel gnathostome characteristic, myelination, suggesting that the acquisition of these DSO‐Gs before gnathostome diversification could have contributed to the acquisition of the novel characteristic in gnathostomes. We expect that our list of DSO‐Gs in each lineage will contribute to a better understanding of the relationship between genetic changes and novel characteristics in the evolution of vertebrates.

## Author Contributions

Hirofumi Kariyayama, Takeshi Kawashima, Haruka Ozaki, and Hiroshi Wada designed the study. Hirofumi Kariyayama and Hiroshi Wada designed and configured the experiments. Hirofumi Kariyayama conducted the experiments. Hirofumi Kariyayama analyzed the data and developed the bioinformatics methods. Hirofumi Kariyayama, Takeshi Kawashima, Haruka Ozaki, and Hiroshi Wada interpreted the data. Hirofumi Kariyayama, Takeshi Kawashima, and Haruka Ozaki prepared figures and wrote the manuscript. All the authors have read and approved the final manuscript.

## Supporting information

Supporting information.

Supporting information.

Supporting information.

Supporting information.

Supporting information.

Supporting information.

## Data Availability

Raw reads of the RNA‐seq data are available from the DDBJ Sequence Read Archive (DRX577178‐DRX577182). The code and data are available at FigShare (https://doi.org/10.6084/m9.figshare.24116610.v1).
