# Supplementary material for: Domain‐Shuffling in the Evolution of Cyclostomes and Gnathostomes
Source: J Exp Zool B Mol Dev Evol. 2024 Dec 4;344(2):59–79. doi: 10.1002/jez.b.23282 (PMC11788884; doi:10.1002/jez.b.23282)
Supplement: Supplementary file 1 — Supporting information. [file JEZ-344-59-s002.docx]

**Appendix A**

**Filter 3. Search domain pairs in outgroup using additional databases**

We removed the class 2 domain pairs that were falsely classified as class 2 domain pairs of a certain lineage, presumably due to poor taxon sampling or incompleteness of gene models. First, we downloaded amino acid sequence datasets of four protochordate (*Branchiostoma* *floridae*, *B*. *belcheri*, *Ciona* *instestinalis*, and *Oikopleura* *dioica*) from GenBank and Refseq databases (O’Leary et al., 2016), searched for domain hits using HMMER3 (Mistry, Finn, Eddy, Bateman, & Punta, 2013) as described in Section 2.2, and checked for the existence of the class 2 domain pairs.

InterPro Application Programming Interface (InterPro API) was used (Paysan-Lafosse et al., 2023) to search for domain architectures (InterPro domain architecture, IDA) that include the queried domain pairs from genes in metazoans (by setting Taxonomy ID to 33208). When we found an IDA containing the domain pair, we retrieved a list of genes associated with that IDA in metazoans. If any gene from outgroup metazoans was included in the gene list associated with the IDA, that domain pair was classified as a domain pair also present in the outgroup species.

**Filter 4-a: Filtering of pairs of domains overlapping with each other**

It was determined whether the annotation of one domain in a lineage-specific domain pair overlapped with the annotation of the other domain. Specifically, if the length of the overlapping region was more than 50% of the length of the shorter domain of the domain pair, we defined the domain pair as overlapping. If this overlap was observed in all species within the lineage (vertebrates, gnathostomes, or cyclostomes), that lineage-specific domain pair was excluded from further analysis.

**Filter 4-b: Filtering of domain pairs with lineage-shared overlapping domain annotations**

If a domain, domain x which is composed of a lineage-specific domain pair (domain x, domain y), overlapped with the location of another domain, domain z, and if a domain pair (domain z, domain y) was found in the outgroups, then (domain x, domain y) was considered to have possibly arisen due to nucleotide substitutions rather than domain shuffling. Thus, we separately classified and filtered such lineage-specific domain pairs.

When two domains were overlappingly annotated to the location, we defined such domains as an overlapping-domain-annotation. Here, such domains were limited to cases where the length of the overlapping region was more than 50% of the length of the shorter annotated domain. The overlapping-domain-annotations were searched across all genes containing lineage-specific domain pairs using the R packages tidyverse (Wickham et al., 2019) and fuzzyjoin (Robinson, 2020). To eliminate the possibility of false positives due to chance similarities, we extracted overlapping-domain-annotations which were shared by all species in the lineage. These were referred to as lineage-shared overlapping-domain-annotations. For each lineage-specific domain pair, if a domain pair in which the domains were replaced by a lineage-shared overlapping-domain-annotation was found in at least one outgroup species, the original lineage-specific domain pair was labeled as a domain pair that may have newly arisen due to sequence substitution. Therefore, we excluded such lineage-specific domain pairs from further analysis.

**Filter 4-c: Filtering of domain pairs with lineage-shared overlapping domain annotations using InterProScan**

To prevent missing overlapping domain annotations, the filtering process for lineage-specific domain pairs was repeated using InterProScan. The amino acid sequences of genes containing lineage-specific domain pairs were used as queries to search for domains in their amino acid sequences using InterProScan version 5.6.9 (Jones et al., 2014) with Pfam, CDD, and SMART as databases. Among the detected domains, lineage-shared overlapping-domain-annotations were extracted as described above.

For each lineage-specific domain pair, the domain pair in which one of the domains was replaced by a lineage-shared overlapping domain annotation was used as a query to search for IDA in metazoan amino acid sequences (by setting Taxonomy ID to 33208) using the InterPro API. If the IDA was detected in at least one outgroup species, the original lineage-specific domain pair was labeled as a domain pair that may have newly arisen due to sequence substitution, and it was excluded from further analysis.

**Filter 5. Filtration of domain pairs that contain truncated domains**

Unreliable domain hits in the outgroup of a certain lineage were excluded. We found that some domain hits were truncated or incomplete in the outgroups and assumed that such domains were not functional or convergently appeared in certain lineages. To exclude such unreliable domain hits, we used an additional threshold of independent E value (I-evalue) < 1 (Supporting information Tables 2, 3, and 4). The remaining class 2 domain pairs were defined as the DSO-DPs.

**Filter 6. Searching for gnathostome DSO-DPs from cyclostome genomes**

To further examine the presence of the regions corresponding to the candidate gnathostome DSO genes in cyclostome genomes, we performed a TBLASTN search against the reference genome sequences of cyclostomes using the gnathostome DSO genes as queries (E-value < 1E-5). Among the TBLASTN hits for each gnathostome DSO gene, we (1) scanned for the blast hits in which a hit region overleaped with the region annotated as either domain of the domain pair by 50% or more on the query sequence, (2) listed up the top 5 blast hits with the smallest E-values for each domain of the domain pair, (3) selected the blast hits in which both domains of the domain pair were located on the same cyclostome contig, (4) for each of the selected blast hits, extracted the subsequence on the database sequences (cyclostome genome sequences) and translated the extracted nucleotide sequence into the amino acid sequence using seqkit version 2.8.2 (Shen, Sipos, & Zhao, 2024), and (5) performed a BLASTP search using these translated amino acid sequences as queries against human gene models, with an E-value threshold of 1E-5. If the best BLASTP hit was any human gnathostome DSO gene, the corresponding gnathostome DSO domain pair was regarded as domain pairs potentially missed in cyclostomes.

**Reference**

Jones, P., Binns, D., Chang, H.-Y., Fraser, M., Li, W., McAnulla, C., … Hunter, S. (2014). InterProScan 5: genome-scale protein function classification. *Bioinformatics (Oxford, England)*, *30*(9), 1236–1240.

Mistry, J., Finn, R. D., Eddy, S. R., Bateman, A., & Punta, M. (2013). Challenges in homology search: HMMER3 and convergent evolution of coiled-coil regions. *Nucleic Acids Research*, *41*(12), e121.

O’Leary, N. A., Wright, M. W., Brister, J. R., Ciufo, S., Haddad, D., McVeigh, R., … Pruitt, K. D. (2016). Reference sequence (RefSeq) database at NCBI: current status, taxonomic expansion, and functional annotation. *Nucleic Acids Research*, *44*(D1), D733-45.

Paysan-Lafosse, T., Blum, M., Chuguransky, S., Grego, T., Pinto, B. L., Salazar, G. A., … Bateman, A. (2023). InterPro in 2022. *Nucleic Acids Research*, *51*(D1), D418–D427.

Robinson, D. (2020). *fuzzyjoin: Join Tables Together on Inexact Matching*. Retrieved from R package version 0.1.6, https://CRAN.R-project.org/package=fuzzyjoin

Shen, W., Sipos, B., & Zhao, L. (2024). SeqKit2: A Swiss army knife for sequence and alignment processing. *IMeta*, *3*(3), e191.

Wickham, H., Averick, M., Bryan, J., Chang, W., McGowan, L., François, R., … Yutani, H. (2019). Welcome to the tidyverse. *Journal of Open Source Software*, *4*(43), 1686.
