# Supplementary figures and images for: Domain‐Shuffling in the Evolution of Cyclostomes and Gnathostomes
Source: J Exp Zool B Mol Dev Evol. 2024 Dec 4;344(2):59–79. doi: 10.1002/jez.b.23282 (PMC11788884; doi:10.1002/jez.b.23282)

A

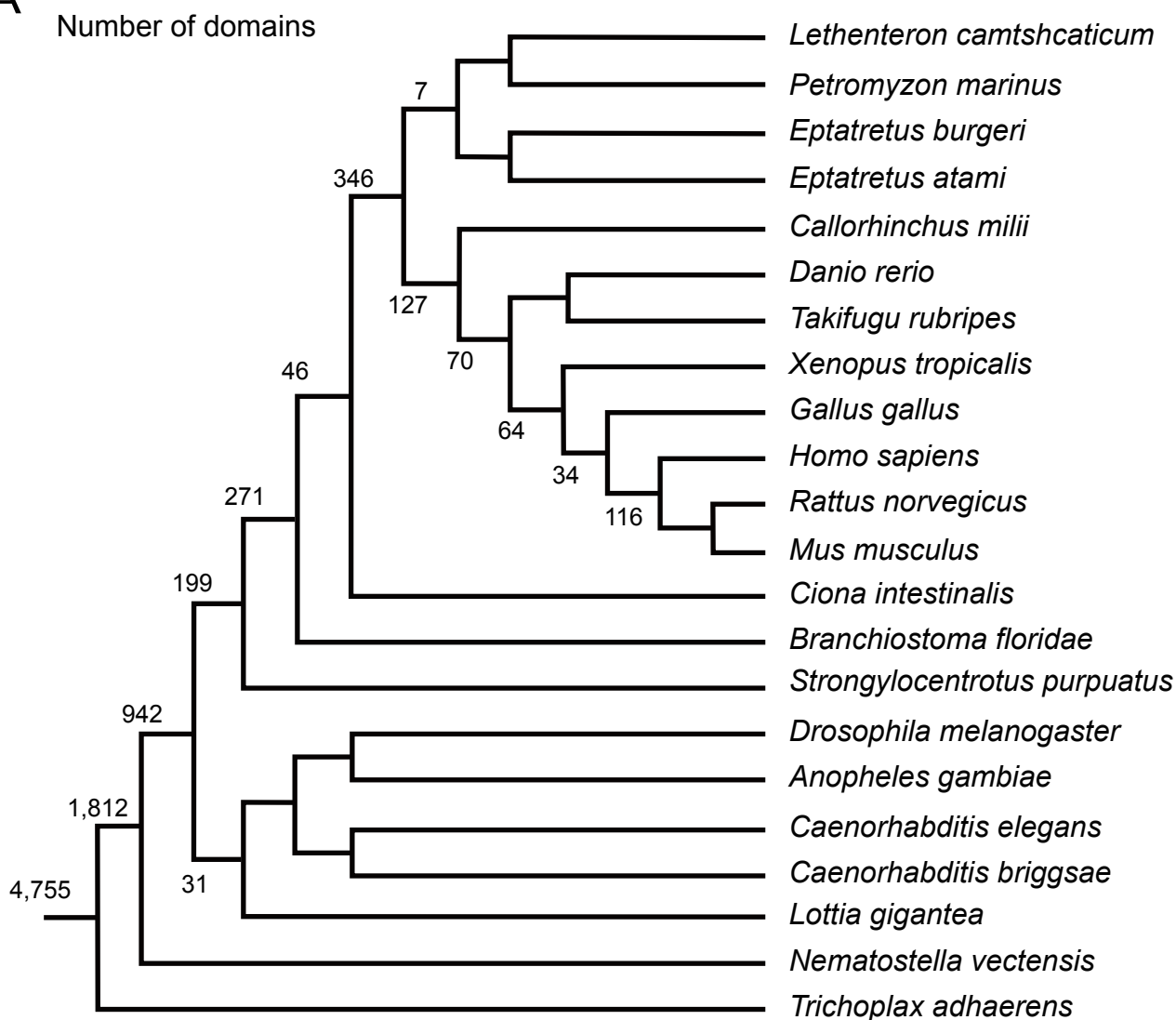

B

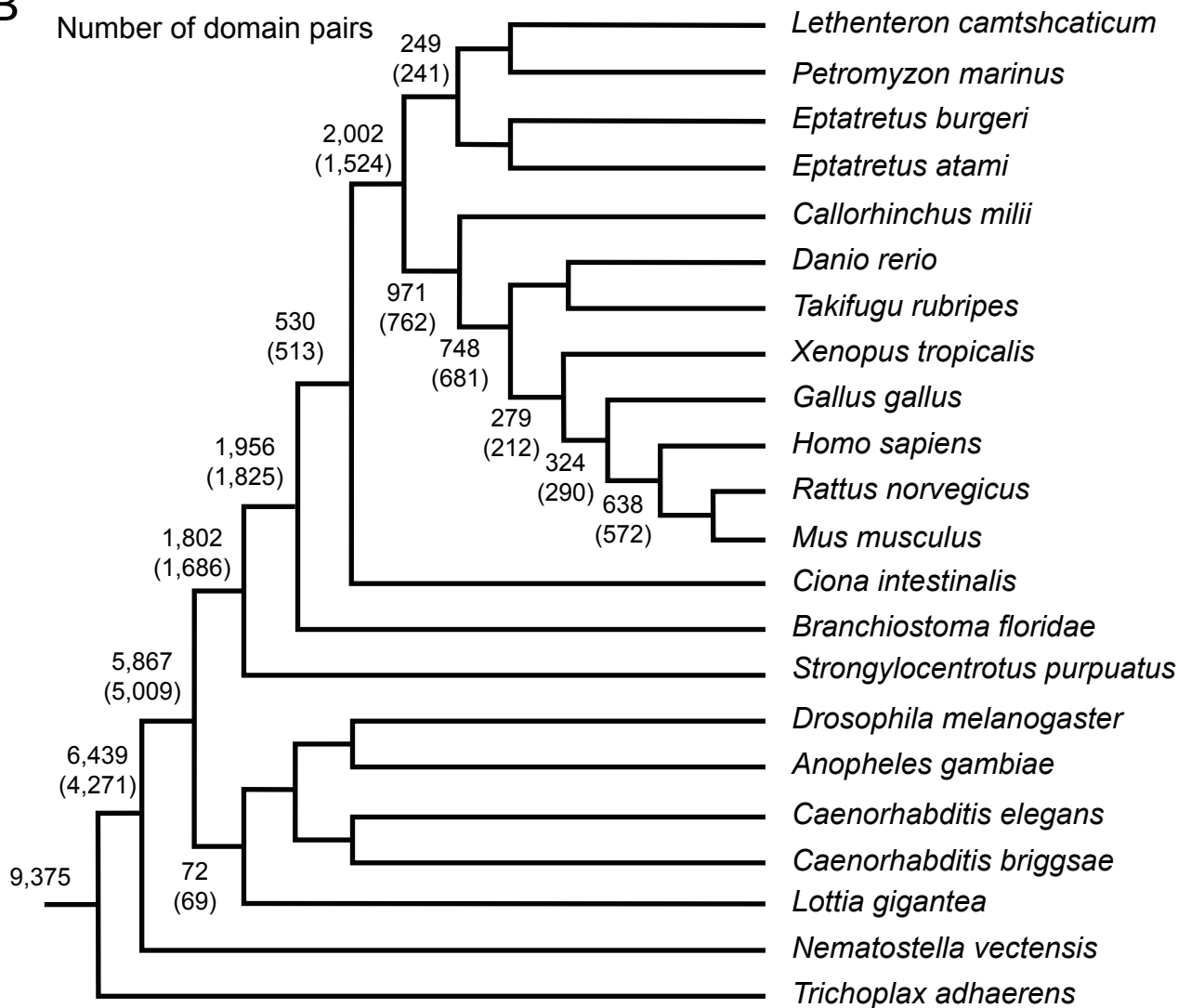

Supplement: Supplementary file 3 — Supporting information. [file JEZ-344-59-s001.pdf]

A

## Domains

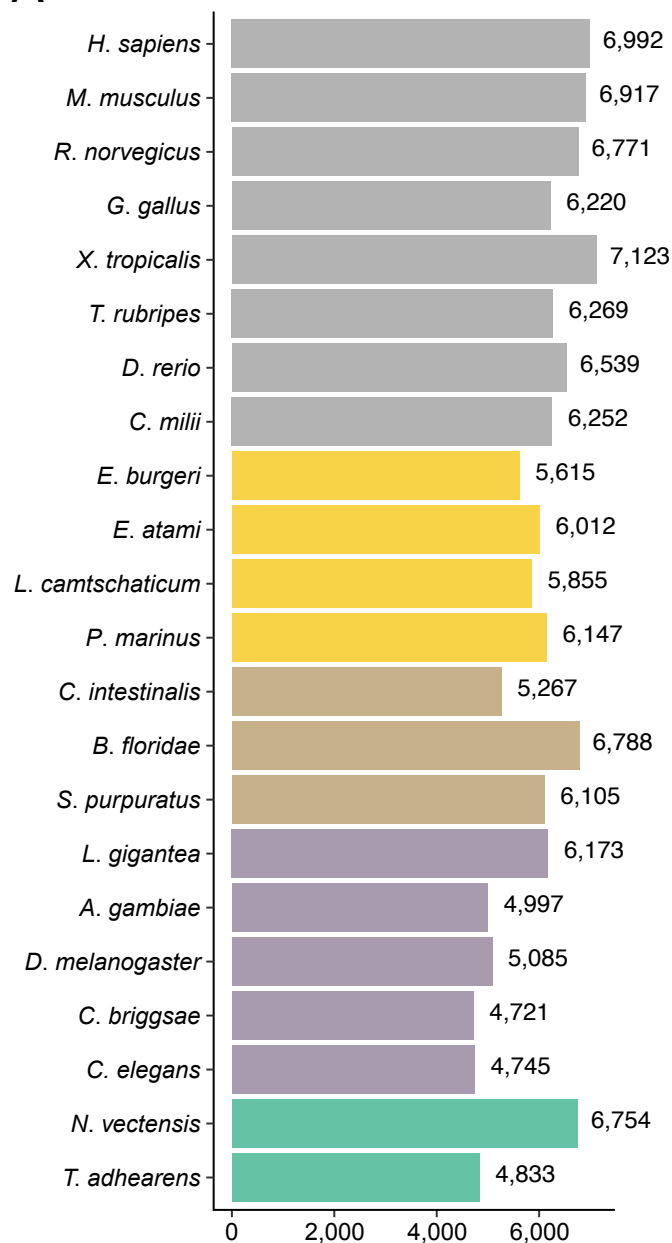

B

## Domain pairs

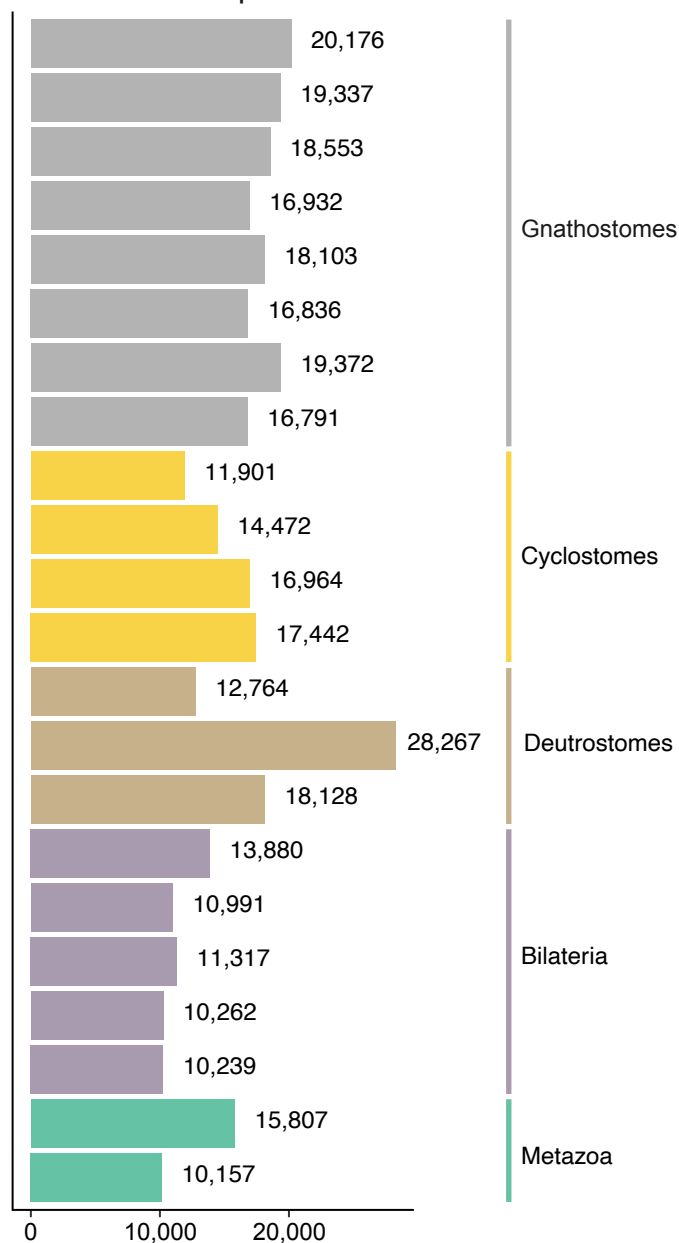

Count

Supplement: Supplementary file 4 — Supporting information. [file JEZ-344-59-s004.pdf]
