# Supplementary material for: Domain‐Shuffling in the Evolution of Cyclostomes and Gnathostomes
Source: J Exp Zool B Mol Dev Evol. 2024 Dec 4;344(2):59–79. doi: 10.1002/jez.b.23282 (PMC11788884; doi:10.1002/jez.b.23282)
Supplement: Supplementary file 5 — Supporting information. [file JEZ-344-59-s005.pdf]

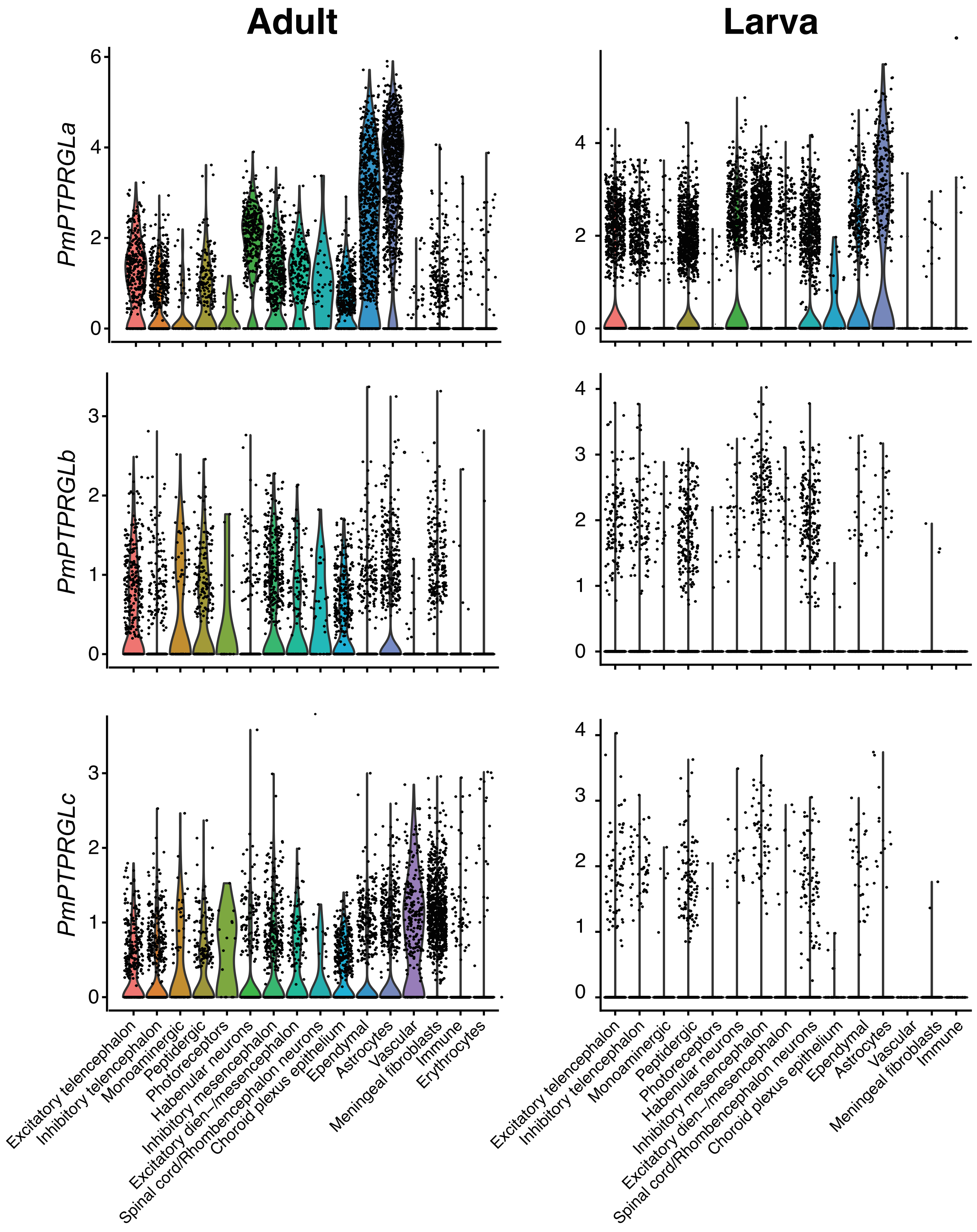

Adult
Larva
PmPTPRGLa
PmPTPRGLb
PmPTPRGLc
0
2
4
6
0
1
2
3
4
Excitatory telencephalon
Inhibitory telencephalon
Monoaminergic
Peptidergic
Photoreceptors
Habenular neurons
Inhibitory mesencephalon
Excitatory dien-/mesencephalon
Spinal cord/Rhombencephalon neurons
Choroid plexus epithelium
Ependymal
Astrocytes
Vascular
Meningeal fibroblasts
Immune
Erythrocytes
