# Supplementary material for: Domain‐Shuffling in the Evolution of Cyclostomes and Gnathostomes
Source: J Exp Zool B Mol Dev Evol. 2024 Dec 4;344(2):59–79. doi: 10.1002/jez.b.23282 (PMC11788884; doi:10.1002/jez.b.23282)
Supplement: Supplementary file 6 — Supporting information. [file JEZ-344-59-s003.docx]

**Supplementary Materials**

**Supporting Information Figure 1. The number of domains and domain pairs in each lineage.**

(A) The number of domains shared among at least two organisms. (B) The number of domain pairs shared among at least two organisms. The numbers in the brackets show the number of domain pairs identified as class 2.

**Supporting Information Figure 2. The number of domain pairs in each animal.**

(A) The number of domains is included in the gene models. The x-axis shows the number of domains, and the y-axis shows the species. (B) The number of domain pairs is included in the gene models. The x-axis shows the number of domain pairs, and the y-axis shows the species.

**Supporting Information Figure 3. The expression of *PmPTPRGL* genes in the lamprey brain atlas**

The expression distributions of the sea lamprey *PTPRG-like* genes in brain atlas. The x-axis shows cell types annotated in the datasets. The y-axis shows the normalized expression amount of *PmPTPRGL* genes in each cell. Pm, *Petromyzon marinus*.

**Supporting Information Table 1. The list of the gene models used for domain search**

**Supporting Information Table 2. The filter result for the domain pairs found only in the gene models of all gnathostomes and any cyclostomes**

**Supporting Information Table 3. The filter result for the domain pairs found only in the gene models of all gnathostomes**

**Supporting Information Table 4. The filter result for the domain pairs found only in the gene models of both at least one of the lampreys and the hagfishes**

**Supporting Information Table 5. The primer list**

**Supporting Information Table 6. The list of genes used in the phylogenetic analysis**

**Supporting Information Table 7. GO analysis about the vertebrate DSO-Gs**

**Supporting Information Table 8. Domain pair search for the vertebrate DSO-DPs from the lamprey transcriptome data**

**Supporting Information Table 9. The number of homologous sequences to the vertebrate DSO-Gs in the lamprey transcriptome data**

**Supporting Information Table 10. The number of vertebrate DSO-DPs in the lamprey gene models from the genome data and the lamprey transcriptome data**

**Supporting Information Table 11. Blast best hit sequence of the lamprey DSO genes against the Swiss-Prot database**

**Supporting Information Table 12. GO analysis about the gnathostome DSO-Gs**

**Supporting Information Table 13. The number of sequences including the cyclostome DSO-DPs for the lamprey transcriptome data**

**Supporting Information Table 14. Domain pair search for the cyclostome DSO domain pairs from the lamprey transcriptome data**
